# Supplementary material for: Multiple patterns of persistent inward currents with multiple types of repetitive firings in medullary serotonergic neurons of mice: An experimental and modeling study
Source: PLoS Comput Biol. 2025 Apr 9;21(4):e1012918. doi: 10.1371/journal.pcbi.1012918 (PMC11981129; doi:10.1371/journal.pcbi.1012918)
Supplement: Attachment — Submitted filename: Point to Point Respones to Reviewers Comments.docx [file pcbi.1012918.s005.docx]

**Reviewer #2: Thank you for the modifications to the paper. The revised version is now clearer than the previous one.
Response:** Thanks for the reviewer’s summary.

**I understand that you want to stand by your version of the manuscript without conducting additional computational experiments, and that you wish to save them for a future paper. This would have added significant value to the current paper and strengthened the message.**

**Response:** Thank you for your valuable feedback. We understand the importance of the additional computational experiments and agree that they could enhance the current paper. However, focusing on the core findings of this study could provide a clearer message for our readers.

**I still notice some recurring inconsistencies with the figure and trajectory labels. We are converging toward a polished version, but wherever you show a trajectory, the I-V axis is needed.**

**Response:** Thanks for the reviewer’s diligent work toward a polished version of this manuscript. However, as we have addressed the axis issues in the last version of our responses to the reviewers, the persistent inward current (PICs) is a function of two variables, the membrane potential and time. Therefore, it is appropriate to express PICs in axes of both voltage and time, and this was what we had done in the Figures 3&6 in the last revised version. However, in this revised version, we have added the ascending and descending arrows to the voltage and current ramps to show the full ranges of the stimulus which correspond to the changes in the PICs trajectory.

**Minor Review**

**-** **Figure 2A: The trajectory I vs V is still missing an axis. There are V vs time and I vs time plots, but the trajectory is never associated with the I-V axis.**

**Response:** Actually, PICs (I) vs V and PICs (I) vs time are shown in the left panel of Fig. 2A1. These calibration bars are applicable to all panels (right and left) of Fig. 2A. The so-called PIC trajectory includes PICs in both left and right panels of Fig. 2A, and the right panels are used to show the mirrored trajectory of PICs (folded from midline) with the same calibration bars in the left panel of Fig. 2A1. It is unnecessary to add extra calibration bars to the right panels. This is a traditional method to express PICs.

**- Figure 3: The axes are incorrect. According to your axes, the current can move backward in time (to the left). Once again, a trajectory is defined in I-V space. Time is the variable that is used to move along the axis (indicated by the arrow). The bi-ramp consists of the increase and decrease of V, which is why you cannot align a trajectory with just half of the stimulus. It should be the same as in Figure 2, where the stimulus is shown in full.**

**Response:**  We have explained the axis issues in the above responses, therefore there is no need for us to repeat them here. But we want to emphasize that we can definitely show the PICs with just half of the stimulus, depending on the issues we are focusing on. In Fig. 3 (Fig. 6, as well), we investigated the PIC patterns and firing types with modulation of gNaP, therefore the mirrored PICs and mirrored firing frequency are required to address these issues.

We have added the ascending and descending arrows to the ramp voltage (Fig. 3A) and ramp current (Fig, 3B) to represent the full ranges of the stimulus, which correspond to the ascending and descending changes in the PIC trajectory and firing frequency, respectively.

**Figure 6: There is the same inconsistency with the axes. The current cannot move backward in time. This should be represented in I-V space, with the arrow indicating the progression along the time axis.**

**Response:** The same as we have done in Fig. 3, we have added the ascending and descending arrows to the voltage and current ramps in panels A and B of Figure 6.

**- Methods: Thank you for modifying the notation for the m and h variables in the conductance-based model. However, there is still an underscore between the m or h and the subscript of the current name (e.g., m_NA). The underscore must be removed and the subscript must stay. This formatting should be updated for each current.**

**Response:** Thanks for the reviewer’s advice. We have removed the underscore after the activation variables throughout the manuscript and figures and maintained the subscript format uniformly for the current names. Please see the Methods and Figures 4A and 7A.
