# Supplementary material for: Multiple patterns of persistent inward currents with multiple types of repetitive firings in medullary serotonergic neurons of mice: An experimental and modeling study
Source: PLoS Comput Biol. 2025 Apr 9;21(4):e1012918. doi: 10.1371/journal.pcbi.1012918 (PMC11981129; doi:10.1371/journal.pcbi.1012918)
Supplement: Attachment — Submitted filename: ReviewerComments.docx [file pcbi.1012918.s001.docx]

Summary:

This work looks at the mechanistic impacts of different ion channel and morphology parameters on output electrophysiology, in particular persistent inward currents. While uncovering mechanistic effects on electrophysiology is interesting, the paper lacks some key details and contexts for non-expert readers to understand the importance of the work, e.g.:

Major:

For a non-expert in PICs, figure 1 is very difficult to follow. What are the orange and blue dashed lines? Where are the scale bars? What is a-PIC vs d-PIC? This first panel is an important setup for the rest of the paper, and the authors can do better at annotating and explaining PICs to make it easier for the reader. Panels B and C could also benefit from better labeling.

How the base model was developed and fit to experimental recordings is not well described and is missing from the results. Were there subtype models that fit to each subtype of PIC/spiking? How well does it capture the data? The authors jump to describing what happens when you play around with the PIC parameters without even convincing the reader that the model is a sufficient model to begin with. Moving figure 10 to be presented earlier in the paper results could help clarify these aspects.

Related to the above, why set NaP/Ca1.3 conductances to zero when manipulating the other parameter? It is unclear if the model still captures the experimental data it was fit to when doing this, and so it is unclear if manipulations on top of this will generate reliable results.

Can the authors demonstrate that the investigated ranges of conductance and kinetics in the Na-PIC and Ca-PIC models are within physiological ranges for these channels? The changes in dendritic length appear to be particularly large (so not surprising that the effects were dramatic).

Why is clockwise vs counterclockwise hysteresis important? The impact that it might have on spiking is not described. Also, why is it important in the context of locomotion?

Is the total Ca-PIC conductance kept fixed when increasing dendritic length? Otherwise, effects of distribution are conflated with effects due to increased total conductance.

Much of the discussion could be sharpened – a lot of it just re-states the results without adding enough contextualization of the results.

Minor:

Line 88: typo – “unknow”.

Figure 1B: What do the red vs blue dots mean?

Figure 1C: Panel A shows 6 types of PICs, but the proportions in this panel are only shown for three based on only the ∆V criteria. Probably to hint at overlap with firing type (∆V<0 vs ∆I<0). If the PIC and firing rate experiments were done on the same neurons, what are the actual overlaps between PIC types and firing types?

Lines 157-158 – do the authors mean to say “… soma, dendrites, axon initial segment, and axon”?

Line 283: type – “dominate” instead of “dominant”
