# Supplementary material for: Multiple patterns of persistent inward currents with multiple types of repetitive firings in medullary serotonergic neurons of mice: An experimental and modeling study
Source: PLoS Comput Biol. 2025 Apr 9;21(4):e1012918. doi: 10.1371/journal.pcbi.1012918 (PMC11981129; doi:10.1371/journal.pcbi.1012918)
Supplement: Attachment — Submitted filename: Point to Point Respones to Reviewer2s Comments-Final.docx [file pcbi.1012918.s003.docx]

**Reviewer #2: **Summary ****

**The manuscript investigates the role of persistent inward currents (PICs), such as sodium and calcium channel currents, in serotonergic neurons of the medulla. It combines experimental and modeling techniques to explore the role of these currents in the different firing activities of these neurons. The experimental approach revealed different patterns of PICs and their associated neuronal firing activities. The computational approach helps modulate the currents by adjusting conductance and kinetics, specifically by tuning half-activation and ion channel gating.**

**Response:** Thanks for the reviewer’s concise summary of this study.

****Global comments****

**I believe that the paper presents interesting findings. However, the authors need to make major revisions:**

**I. The authors use specialized vocabulary that assumes prior knowledge, such as "hysteresis" (mentioned in the abstract and text), terms like "counterclockwise," and phrases such as "manifold types of repetitive firing" (in the title and text). The authors need to improve how they convey their message to a broad audience by ensuring that each concept is clearly defined or well-introduced. I strongly recommend improving this aspect of the manuscript. The introduction should provide clear definitions of important concepts. Additionally, the title and abstract should be reformulated to make them more accessible and engaging to a wider audience.**

**Response:** Thanks for the reviewer’s suggestions. We have revised the Title and Abstract of the paper, reducing the use of technical terminology. Additionally, we have included explanations of technical terms such as “clockwise,” “counterclockwise,” in the Introduction section. Please see the revised Introduction.

**II. In contrast, some sections of the results (especially those related to Figures 8 and 9) could be summarized more concisely. The authors often describe figures in great detail, but there is a lack of perspective linking the computational experiments to biological neuronal behaviors. This often leads to the question, “And so what?” The authors must ensure that, whenever a computational experiment is conducted, the underlying logic behind it is clearly explained. Additionally, the implications of the results should be clearly conveyed and discussed.**

**Response:** Thanks for the reviewer’s advice. We have revised the descriptions of Figures 9 and 10 in the new version of this manuscript. In the Result section, we have added the relevance of the model to physiological experiments and explained the rationale of altering the parameters related to ion channels. Based on the reviewers' comments, we have also rewritten the Discussion section of the paper.

**III. I do not understand the choice of the terms “Ca-PIC” and “Na-PIC.” These currents seem to have already been described. If Ca-PIC represents the same thing as CaV1.3/CaL (mentioned on line 273, 339, 675), using different terms makes the message less clear. At the end of the text, the authors attempt to link them. If they are the same, the authors should use the most commonly accepted conventions, such as CaV1.3/CaL or NaP, and briefly describe how they work.**

**Response:** We are sorry for confusing the terms of NaP and Cav1.3. Based on the purpose of the present study, we have designated the "persistent sodium channels" as NaP and "persistent calcium channels" as CaL throughout the manuscript with brief definitions in revised manuscript.

**IV. The flow of the text in English needs to be improved to enhance the quality of the manuscript. I suggest consulting a scientific writing expert to help improve the overall structure and phrasing of some sentences. There are many repetitions, and the text length could be significantly reduced.**

**Response:** Thanks for the reviewers' thoughtful comments. We have carefully reviewed the manuscript and improve the English writing in the revised manuscript.

**V. I also have a general comment about the figures. The quality of all figures needs to be revised. They should be presented in vector format to ensure clarity. Many figures are missing axis labels, color bar labels, and x-scale information.**

**Response:** Thanks for the reviewer’s kind advice. In the new version of the manuscript, we have uploaded high-resolution images and added axis labels, color bar labels, and x-scale information to the figures. Please also note that the figures were uploaded in high-resolution Tif format. But the resolutions were reduced automatically by PLOS-CB website when converted into PDF file. However, readers could download the high-resolution images by clicking the links on the top right side of the figures.

**VI.The authors use NEURON. The code containing the differential equations, as well as the code to reproduce the figures, must be made available on GitHub or a similar repository.**

**Response:** Yes, as usual we will make our code publicly available on <https://github.com/Cheng-ECNU/5-HT-model-PIC> after the paper is published.

**I found a number of issues that should be addressed before publication. Please see the complete report on the following pages. If any comments are expressed as questions, you should modify the text to make it clear on the first reading, so readers do not have to search for answers or look for missing explanations about a concept.**

**** Precise comments ****

***Title**

**1. Line 1: The title needs to be changed. It does not convey a clear message. "Manifold types" is not a commonly used term in neuroscience. Similarly, in the abstract, "manifold types of repetitive firings" is not a standard term. If this phrase is used in the abstract, it should be defined.**

**Response:** We have revised the title for clearer message (Please see line 1-3)

**Abstract**

**2. Line 21: Clarify the phrase “role in the locomotor system.”**

**Response:** We have changed " Persistent inward currents (PICs) play multifunctional roles in locomotor system. " to " Persistent inward currents (PICs) play a crucial role in regulating neuronal excitability." (Please see line 19-20)

**3. Line 29: The phrase “P3-6” does not communicate effectively to the computational neuroscience community.**

**Response:** “P3-6” stands for postnatal day 3-6. We change the text. (Please see line 27)

**4. Line 32: It is unclear what is meant by “patterns of PIC and repetitive firings.**

**Response:** Thanks for reviewer’s comments. We have rephrased this sentence as “PICs in 5-HT neurons were classified into six patterns based on their trajectory and activation voltage, and repetitive firings were categorized into three types according to neuronal response to ramp current injection.” (Please see line 30-32)

**5. Line 34: This is presented as the second result of the paper, but in the results section, the Na-PIC analysis is discussed first. This is inconsistent.**

**Response:** We have changed the order of results in the abstract, presenting the NaP first, followed by the CaL. (Please see line 34-41)

**6. Line 35: Avoid abbreviations in the abstract. “Vs” is not a standard term.**

**Response:** We had rewritten V_1/2_ as "half-activation voltage" and V_S_ as " slope of activation." (Please see line 38-39)

**7. Line 39: The phrase “****counterclockwise to clockwise” is not common or understandable to a broad audience. It requires reading the results section and referring to Figure 1 to comprehend the concept.**

**Response:** Hysteresis is special term to describe the PIC properties in response to voltage ramp command. However, in order to make this PIC property easily understandable, we simplify the description of PICs and change the “hysteresis” to “trajectory”. We have revised this sentence as “Increasing S_gate_ changed trajectory of PICs from counterclockwise to clockwise and firing types from asymmetrical to symmetric types induced by current ramp injection.” (Please see line 35-37). In addition, we add a description of “counterclockwise to clockwise” to Introduction. (Please see line 78-83)

**8. Line 53: Refer to comment 7 and the general comment. This concept should be clearly introduced and explained.**

**Response:** We have changed "hysteresis" to " trajectory" (Please see line 50-55). We also add an explanation of “counterclockwise to clockwise” to the Introduction. (Please see line 78-83)

*** Introduction**

**9. Line 80: The phrase “manifolds types of repetitive firings” is not a widespread concept in neuroscience. It even gives the impression to be AI-generated, as it does not show up in Google searches as an existing phrase.**

**Response:** We agree with the reviewer that “manifold types” is not an appropriate term to describe the different types of repetitive firing. For this reason we use “multiple types of repetitive firing” or “three types of repetitive firing” to replace the “manifolds types” in revised manuscript. (Please see line 86)

**10. Line 81: What does “underlying the generation of staircase PICs” mean? Please clarify.**

**Response:** “mechanism underlying the generation of staircase PICs” means mechanism responsible for induction of staircase-like PICs. The staircase PIC is a special shape of PICs which is composed of two staircase-like inward currents. This type of PICs was first reported in 5-HT neurons in our previous study (Cheng et al. 2020). We rewrite this section as “mechanism underlying staircase PICs in medullary 5-HT neurons, a special shape of PICs composed of two staircase-like inward currents.”

(Please see line 87-90)

**11. Lines 94-95-98: Refer to comment 7. The concept is mentioned three times in four lines but remains very unclear. In the revised manuscript, this must be clearly addressed.**

**Response:** As mentioned above, hysteresis is special term to describe the PIC properties in response to voltage ramp command. However, in order to make this PIC property easily understandable, we simplify the description of PICs and change the “hysteresis” to “trajectory”. In addition, we also add a description of “counterclockwise to clockwise” and classification method for PIC trajectory to Introduction section. (Please see line 78-83)

*** Results**

**12. Line 139: Figure 1:**

**The quality of the figure is very poor. Everything is pixelated, even when downloaded in .tif format. There are no units or labels. Clear vertical and horizontal scales must be added, as well as appropriate labels. The traces should be in SVG, EPS, or PDF format for better quality.**

**Response:** Thanks for reviewer’s comments. In revised version, we have uploaded high-resolution images to the website. As reviewers’ suggestion, Figure 10 is move to the first place as Figure 1, and the old Figures 1-9 are changed to Figure 2-10 in the new version. We also add the scale bars and labels to the Figure. 2 and rewrite the legend. (Please see line 175-192)

**13. Line 100-139: Overall Results Section for Figure 1:**

**The result section associated with this figure needs to be modified. Here is a potential suggestion:**

**(i) What is a PIC?**

**(ii) How are PICs categorized experimentally? Explain the method and terminology used.**

**(iii) Include a table or summary of the six patterns with the different values taken by the key categorizing parameters.**

**The overall flow and concepts are not enough clearly defined while the text relies on these definitions.**

**Response:** Thanks for the reviewer’s advice.

We add a section to Results titled “Classification of PICs patterns and firing properties”, which described the measurement of PICs, classification of patterns and firing types, and structure of 5-HT neuron model. (Please see line 104-139)

**14. Line 105: There is no explanation of what a-PIC and d-PIC, Vonset, ∆V, etc., are. The definitions of the six patterns need to be improved. The diagram shown in Figure 10B should appear in Figure 1, along with Figure 10C to explain what ∆V and ∆I are. Without this, the text is unclear, and readers cannot easily distinguish between the patterns.**

**Response:** Thanks for the reviewer’s advice.

We have moved Figure 10 to Figure 1 and added new section “Classification of PIC patterns and firing types” to Results in revised manuscript, where we described the measurement of PICs, classification of and firing types, and structure of 5-HT neuron model. In this section, we also add the definitions of parameters such as a-PIC, d-PIC, V_onset_, V_offset_, I_onset_, I_offset_, ∆V and ∆I. (Please see line 109-116)

**15. Line 116: A reference to the different traditional methods for classification is missing.**

**Response:** Thanks for the reviewers' reminders. We have added the references to the manuscript. (Please see line 82)

**16. Line 153: This seems like a conclusion or a transition to the next paragraph. It seems like the title should appear earlier in the flow of the text.**

**Response:** Thanks for the reviewers' comments. We have added the title “Contributions of NaP and CaL to PIC patterns and firing types” to Results. (Please see line 194)

**17. Line 158: “Conductances” should be plural.**

**Response:** Thanks for the reviewers' reminders. we have corrected it. (Please see line 121)

**18. Line 159: Add a link to the methods section.**

**Response:** We have added “(see Methods)”. (Please see line 200)

**19. Line 165: The term “staircase PIC” is unclear and not defined or introduced properly.**

**Response:** We have added the definition of staircase PIC to the Introduction. (Please see line 87-90).

**20. Line 178: Why is** **gNa-PIC not tested below 100%, like at 50%? Why does it go up to 500%? The axis does not need to go to 600% if the data stops at 500%. Why not use absolute numbers (1, 2, 3, etc.) instead of** **percentages?**

**Response:** Yes, gNa-PIC could be tested below 100% such as 50%. But the key point of gNa-PIC modeling is to predict trend of changes in PIC patterns (∆V) and firing types (∆I) when gNa-PIC was altered. As shown in new Figure 3, gNa-PIC-induced changes in V_onset_, V_offset_, I_onset_, and I_offset_ exhibited highly linear relations (R^2^>0.9). Therefore, based on the linear regression lines in Figure 3C-F, changes in PIC patterns and firing types could be easily predicted within a wide range of gNa-PIC alteration from 0% to 600%.

To make figures looking better (regression lines remain within the axis) we extended axis to 600% even the data stop at 500%.

Yes, the absolute values could be used for gNa-PIC. But to make the results comparable with gCa-PIC, we use percentages for data description in the figures.

**21. Line 208: For the blockage of Ca-PIC, specify that you set the conductance to zero.**

**Response:** Thanks for the reviewer’s advice. We had added “(gCaL=0)” after “blockage of CaL”. (Please see line 249)

**22. Lines 218-219: You should refer to a figure or explain the concept.**

**Response:** Thanks for the reviewer’s advice. We rewrite this paragraph as “These results indicated that increasing the V_S_ of NaP did not change the counterclockwise trajectory (Fig. 4F and G) of the PICs and the ΔV (Fig. 4D), nor did it change the firing type of the neuron (Fig. 4E). These results further suggested that V_S_ of NaP channels had little impact on the patterns of PICs and firing types of 5-HT neurons.” (Please see line 257-261)

**23. Line 221: For subpanel 3C, consistency is needed:**

**• Either make the line and the label 'Vs=3mV' in the same color.**

**• Or make the line in the associated color but keep the label in black. The current mix of color codes between Vs=2 and 7 is confusing.**

**Response:** Thanks for the reviewer’s advice. We remove the line for V_S_ = 2 and add the line for V_S_ = 3, ensuring that the line and the label colors are consistent. (Please see Fig. 4A, B and C)

**24. Line 221: Figure 3C is essentially your equation from line 698. It would make more sense to have it in panel A, as it involves adjusting Vs in the computational experiment. Then, show panels A and B.**

**Response:** Thanks for the reviewer’s advice. In the new version of manuscript, we adjust the order of figure panels and put the Boltzmann equation from line 698 to the panel A as the reviewer suggested. Previous Figure 3 is changed to Figure 4 in the new version (Please see Fig.4 A, B and C).

**25. Line 221: In Figure 3A, use a lowercase 's' for seconds: “1 s” instead of “1 S.”**

**Response:** Thanks for reviewer’s reminder. This mistake is corrected in Figure 4.

**26. Line 221: I would also suggest that in Figures 3D, 4C, and 5C, the horizontal axis be placed at the bottom of the panel, not at the top. This will make the graphs more consistent with the other three panels.**

**Response:** This is a good suggestion. We have placed the horizontal axis at the bottom of the panels. (Please see Figures 4D, 5C, and 6C)

**27. Line 221: Consistency is needed in the labels. In Figure 3, you fully describe 'm activation,' but in Figure 4, you just say 's_gate.'**

**Response:** We agree with the reviewer. In fact, the S_gate_ referred to the probability of NaP inactivation, ranging from 0 to 1. Traditionally, this state variable is designated by h or s. However, to keep it consistent with Dr. Powers’ group, where the S_gate_ equation and code came from, we decided to use the same symbol S_gate_ in this study. We have added the sentence “The S_gate_ referred to the probability of NaP inactivation, ranging from 0 to 1” to the text. (Please see line 281-282)

**28. Line 222: Clarify whether “recorded” refers to data simulated by the code or recorded in an experiment. This should be made clear immediately. I suggest reordering the figure subpanels for clarity.**

**Response:** In general, data from experiments were referred to as “recorded”, while simulation data from modeling were called “induced” or “evoked”. For example, “PICs were induced with……”; “Receptive firings were evoked with ……”. We have clarified this confusion throughout the manuscript.

**29. Line 240: Indicate the method again.**

**Response:** We have added " (see methods)"to the text. (Please see line 282)

**30. Lines 250-251: This section describes the figure but does not explain the implications for neuronal activity.**

**Response:** We rewrite this paragraph as “The above results suggested that the S_gate_ of NaP contributed to regulation of counterclockwise trajectory of PICs. Increasing S_gate_ prolonged the duration of firing in descending phase of ramp current thus increasing the excitability of neuron.” (Please see line 297-300)

**31. Line 252: Replace the phrase “it’s worth noting” with something more formal, as this expression is not appropriate for a scientific article.**

**Response:** We change "it's worth noting" to " It is noted that." (Please see line 296)

**32. Line 275: Maintain consistent grammar; avoid mixing past and present tenses.**

**Response:** We correct the grammar of the sentences for consistency. Thanks! (Please see line 319-321)

**33. Line 280: Indicate how** **gNa is set to 0 (gNa=0).**

**Response:** It should be gNaP. “gNaP is set to 0” means that the maximum conductance of NaP was set to 0 in the model. We add this explanation to the text. (Please see line 325)

**34. Line 282: How did you decide on the range of changes for gCa-PIC? Why is it different from gNa-PIC?**

**Response:** The range of changes for gCaL was similar to that for gNaP in the simulations. This range of conductance in the CaL and NaP models was based on previous modeling studies as well as experimental data. Usually, the alteration ranges of conductances are set to 100-200% of control values (control was defined as 100% in the present model) in previous modeling studies (Zhang and Dai 2020; Zhang et al. 2022). In this study, however, we increased conductances up to 500% in some cases in order to predict the trends of modulatory effects of CaL and NaP on PIC patterns and firing types. In fact, previous studies have reported that moderate intensity chronic exercise induced ~70% increase in the amplitudes of CaL and NaP (i.e. conductances) in spinal neurons (Chen and Dai. 2022), suggesting that CaL and NaP conductances are changeable during exercise and that the degree of changes could depend on the intensity and performance of the exercise. These results have provided a physiological basis for modulating of CaL and NaP. In the present study, we altered PIC conductances in proportion to the experimental data.

We discuss this issue in the “Simulation issues related to the model” in Discussion. (Please see lines 636-655)

**35. Line 312: Why is Vs modulated to adjust the kinetics instead of** **tau? A better justification for the interest and necessity of adjusting the kinetics is missing.**

**Response:** Vs determines the sensitivity of NaP channel to membrane potential while the time constant tau regulates the time for the channel opening. Since NaP is a voltage-gated persistent opening channel, modulating Vs produces bigger effect on PIC patterns and firing types than modulating tau. For this reason, we selected Vs as a major parameter for modulation of NaP kinetics in this study. We have added this content to the Discussion. (Please see line 656-661)

**36. Line 339: You mention that Cav3.1 is distributed along the dendrite, then continue by discussing Ca-PIC. The connection or distinction between Ca-PIC, Cav, and CaL is not clear. The introduction/motivation for investigating the distribution of Ca-PIC is insufficient.**

**Response:** Thanks for the reviewer’s comments. The calcium current (CaL) that constitutes the PICs is mediated by the L-type calcium channel (also designated as Cav1.3) which distributes in the dendrites of neurons (Hultborn et al., 2003; Lee and Heckman, 2000; Carlin et al., 2000). These experimental result forms the physiological basis for us to distribute CaL in distal dendrite of model and to analyze the effect of dendritic distribution of CaL on PIC patterns and neuronal firing. We add this content to the Results. We also unify the terms for L-type calcium current as CaL throughout the manuscript. (Please see line 382-387)

**37. Line 378: Are you blocking gCa as before?**

**Response:** Yes, the calcium channel was blocked. We have added (gCaL = 0) to the text. (Please see line 434)

**38. Line 387: This sentence seems to restate the idea from line 378 with different words but the same structure. It is unclear why this repetition is necessary.**

**Response:** We have deleted this sentence.

**39. Line 389: What are the biological implications? Modify the text to explain the motivation and implications.**

**Response:** Simulation results showed that both ΔV and ΔI decreased when S_gate_ increased and V_S_ decreased (Fig. 9A3; Fig. 9B3). In this case, the duration of PICs was extended and the repetitive firing was prolonged, leading to an increased neuronal excitability. We have added this sentence to the Results. (Please see line 445-447)

**40. Line 393: In Figure 8, the squares in the maps do not help to visualize the mesh within the parameter space. A more accurate mesh should be used with more points.**

**Response:** In revised manuscript, we change Figure 8 to Figure 9, where we describe the overall effects of modifying two kinetic parameters, S_gate_ and V_S_, on the PIC patterns and firing properties. The squares in the maps illustrate the trend of changes in V_onset_, V_offset_, I_onset_, I_offset_, ΔV and ΔI when altering these two parameters.

**41. Line 393: In Figure 8, the color bar label is missing.**

**Response:** Thanks for the reviewer’s reminder. We have added color bar label to the figure. (Please see Figure. 9)

**42. Line 406: What are the implications for the neuron and the network? Modify the text to explain the motivation and implications.**

**Response:** The L-type calcium channels (CaL) are primarily distributed in the dendrites of neurons and play an essential role in amplifying synaptic currents and regulating neuronal excitability (Binder et al. 2020; Roca-Lapirot et al., 2018; Perrier et al., 2002). In the following simulation we investigated the mixed effects of modulating V_1/2_, V_S_ and dendritic distribution of CaL on the PIC patterns, firing properties and neuronal excitability. We focused on the changes in V_onset_, V_offset_, and ΔV under voltage-clamp mode and the I_onset_, I_offset_ and ΔI under current-clamp mode, respectively. The NaP channels were blocked (gNaP = 0) in the following simulations. We have added the motivation for regulating these parameters at the beginning of this paragraph. (Please see line 455-461).

**43. Line 408: This sentence is almost identical to line 399. Should this be Ionset?**

**Response:** We have removed this sentence. Thanks!

**44. Line 421: Figure 9 needs improvement—the choice of axis and the color bar make the image hard to read and understand.**

**• In A1, hyperpolarization of V1/2 means moving up on the vertical axis but decreasing on the color bar axis.**

**• In B2, a reduction of Ioffset is blue, whereas in A3, a reduction of ∆V is yellow, similar to B3.**

**Response:** Thanks for the reviewer’s comments.

In A1, the color bar axis represented the variation in V_onset_, and this result indicated that hyperpolarization of the V_1/2_ lowered the V_onset_ of PIC.

According to the reviewer’s suggestions, we adjusted the direction of the color bar axis in A3. In the new version of the figure, an increase in ∆V was represented by yellow, while a decrease in ∆V was represented by blue, maintaining consistency with the trend of color changes in A1 and A2. Similarly, we made the same modifications in B3. For details, please see Figure 10 in the new version of the manuscript.

**45. Lines 399-419: Consider condensing the text. For example:**

**• A depolarization of V1/2 is biologically represented/caused by...**

**• A reduction of Vs represents...**

**• A dendritic extension is represented/caused by...**

**These three modifications cause several effects:**

**• A1. Depolarization of Vonset, which implies...**

**• B1.** **Increase of Ionset, which implies... etc.**

**It also needs to be clearer which parameters are maintained, since some channels were blocked in previous experiments. We need to know if these conditions are still in effect or if they have returned to baseline.**

**Response:** Thanks for the reviewer’s kind advice. We have rewritten this paragraph for the results in Figure 10, based on the reviewer’s recommendations. (Please see line 468-489). In testing the effects of CaL modulation on PIC patterns and firing properties, we blocked NaP. We have added (gNaP = 0) to the manuscript.

*** Discussion**

**46. Line 429: The concept of switching from clockwise to counterclockwise must be fully explained in the introduction and briefly reintroduced in the discussion. It is mentioned frequently but is not a standard term, and its implications or utility are not clear. This must be clarified in the revised manuscript.**

**Response:** Thanks for the reviewer’s advice. We have added explanations of specialized terminology in the Introduction section of this manuscript, and added a subtitle "Role of PICs in modulating neuronal excitability and locomotion" to the Discussion section. (Please see line 596-614)

**47. Line 441: The title needs to be clearer. The next one, “Contributions of Ca-PIC to PIC patterns and firing types,” seems almost the same but more general.**

**Response:** We have revised the title to “Impact of distal distribution of CaL on prolonged firing” (Please see line 509)

**48. Line 442: There are typos or strange symbols.**

**Response:** We have deleted it. Thanks.

**49. Line 442: Here, you state that L-type calcium (Ca-PIC) channels are mainly distributed on the dendrites of spinal neurons, but in line 339, you mention Cav3. You introduce Cav3 again in line 658. This needs to be made extremely clear in the revised manuscript. I believe that using the new notation Ca-PIC increases confusion, whereas using the appropriate scientific ion channel name makes it clearer.**

**Response:** We are sorry for confusing the notations of calcium channels. The L-type calcium channel, Cav3.1, Ca-PIC and CaL are all referred to the L-type calcium channels. We have unified the notations as CaL throughout the manuscript.

**50. Line 458: Why is the acronym defined again here? The same happens on line 464.**

**Response:** We have removed the full term here and replaced it with the standardized abbreviation CaL.

**51. Line 491:** **The order of presentation in the discussion is inconsistent with the results. You first presented data on Na-PIC, then on Ca-PIC, but in the discussion, you reversed the order. You start by discussing Figure 7, then return to Figures 2 and 3.**

**Response:** Thanks for the reviewer's comments. In the Discussion section, we started with the most important issue or finding reported in this study, and then followed by other issues based on their importance. This is why we first discussed the impact of distal distribution of CaL on prolonged firing, which was primary finding of this study, and then we discussed the NaP and other issues. The order of presentation in the discussion is different from that in results.

**Methods**

**52. Line 576: What is the content of the intracellular solution? If this is indicated in the following section, include a link.**

**Response:** We have added “(see Solutions)” to the text. (Please see line 697)

**53. Line 587: The phrase “line was tangent to the ascending current trace was defined as the onset of” is unclear. The mathematical description needs to be clearer.**

**Response:** We have revised the original sentence to “The last point where the straight line touched the rising current trace was defined as the onset of PIC, and the corresponding voltage on the voltage ramp at this point was defined as the onset voltage of PIC (V_onset_).” (Please see line 707-710)

**54. Line 625: In Figure 10A, there is no scale bar to quantify the image. Acronyms might be added directly to the image to help understand the purpose of the slice image.**

**Response:** Thanks for reviewer’s good comments. We move Figure 10 to the first as new Figure 1and change Figure 1-9 to Figure 2-10 in revised manuscript. We also add the scale bars and acronyms to the figures. (Please see Figure 1)

**55. Line 625: Figures 10B and 10C are missing units.**

**Response:** Thanks. We have added units to Figures 1B and 1C.

**56. Line 634: The code should be made available on GitHub or ModelDB.**

**Response:** Yes, as usual we will make the code publicly available on <https://github.com/Cheng-ECNU/5-HT-model-PIC> after this paper is published.

**57. Line 650: There is an error in the formatting of the title in the second column of Table 2.**

**Response:** We have corrected this error. (Please see line 760)

**58. Line 658: Do not introduce new conventions for acronyms**.

**Response:** We have standardized the notations for CaL and NaP throughout the manuscript and removed this sentence from the text.

**59. Line 663: In "Vth," the “th” should be a subscript, like other variables (Ith, Em, Rin, etc.). This change should be applied consistently.**

**Response:** We have corrected this error. (Please see line 773)

**60. Line 667: Which parentheses are you referring to?**

**Response:** Thanks. We have corrected this error.

**61. Line 672: The name should be consistent with the maximum conductance or vice versa.**

**Response:** We have standardized the abbreviations throughout the manuscript.

**62. Lines 675-676: Currents were introduced in line 652. Here, a different definition or vocabulary is used for the same concept. Consistency is needed throughout the manuscript.**

**Response:** We sincerely thank the reviewer for kind comments. We have made the notations of channels consistent throughout the manuscript.

**Reference:**

Binder MD, Powers RK, Heckman CJ. Nonlinear Input-Output Functions of Motoneurons. Physiology (Bethesda). 2020;35(1):31-9.

Carlin KP, Jones KE, Jiang Z, Jordan LM, Brownstone RM. Dendritic L-type calcium currents in mouse spinal motoneurons: implications for bistability. Eur J Neurosci. 2000;12(5):1635-46.

Chen K, Dai Y. Chronic exercise increases excitability of lamina X neurons through enhancement of persistent inward currents and dendritic development in mice. J Physiol. 2022;600(16):3775-93.

Cheng Y, Zhang Q, Dai Y. Sequential activation of multiple persistent inward currents induces staircase currents in serotonergic neurons of medulla in ePet-EYFP mice. J Neurophysiol. 2020;123(1):277-88.

Dai Y, Jordan LM. Multiple patterns and components of persistent inward current with serotonergic modulation in locomotor activity-related neurons in Cfos-EGFP mice. J Neurophysiol. 2010;103(4):1712-27.

Hultborn H, Denton ME, Wienecke J, Nielsen JB. Variable amplification of synaptic input to cat spinal motoneurones by dendritic persistent inward current. J Physiol. 2003;552(Pt 3):945-52.

Lee RH, Heckman CJ. Adjustable amplification of synaptic input in the dendrites of spinal motoneurons in vivo. J Neurosci. 2000;20(17):6734-40.

Perrier JF, Alaburda A, Hounsgaard J. Spinal plasticity mediated by postsynaptic L-type Ca2+ channels. Brain Res Brain Res Rev. 2002;40(1-3):223-9.

Powers RK, Binder MD. Persistent sodium and calcium currents in rat hypoglossal motoneurons. J Neurophysiol. 2003;89(1):615-24.

Roca-Lapirot O, Radwani H, Aby F, Nagy F, Landry M, Fossat P. Calcium signalling through L-type calcium channels: role in pathophysiology of spinal nociceptive transmission. Br J Pharmacol. 2018;175(12):2362-74.

Zhang Q, Dai Y. A modeling study of spinal motoneuron recruitment regulated by ionic channels during fictive locomotion. J Comput Neurosci. 2020;48(4):409-28.

Zhang Q, Cheng Y, Zhou M, Dai Y. Locomotor Pattern and Force Generation Modulated by Ionic Channels: A Computational Study of Spinal Networks Underlying Locomotion. Front Comput Neurosci. 2022;16:809599.
