# Supplementary material for: Multiple patterns of persistent inward currents with multiple types of repetitive firings in medullary serotonergic neurons of mice: An experimental and modeling study
Source: PLoS Comput Biol. 2025 Apr 9;21(4):e1012918. doi: 10.1371/journal.pcbi.1012918 (PMC11981129; doi:10.1371/journal.pcbi.1012918)
Supplement: Attachment — Submitted filename: Point to Point Respones to Reviewer2s Comments.docx [file pcbi.1012918.s004.docx]

**Reviewer #2:** 

**The authors have done an excellent job reorganizing the figures and text, which now clearly highlights the quality of the work. However, there are still some major concerns that need to be addressed before the manuscript can be accepted for publication.**

**Response:** Thanks for reviewer’s comments.

**Global comment:**

**I.Definition of trajectory: In my initial review, I pointed out that the use of specialized jargon was making the manuscript difficult to understand. For instance, the term "trajectory" was used instead of "hysteresis," which raises concerns. In the abstract, the term "trajectory" is introduced without sufficient context, making it unclear. Specifically, it is important to clarify: the trajectory of which variable? In which plane? And what does this mean for the** **firing activity?**

**Response:** We have defined the term "trajectory" in the abstract, which refers to the current trajectory generated by bi-ramp voltage injected into the neurons. (Please see lines 31). Please note: PICs stands for persistent inward currents. Therefore, the trajectory of PICs we used in the text undoubtedly means the trajectory of current. What does the trajectory mean for firing activity? We don’t know, yet. This is the purpose of this study to explore how multiple patterns (different trajectories) of PICs generate multiple types of firing.

As shown in Figure 1B, the trajectory of PICs is generated by a bi-ramp voltage injected into the neuron (bottom). Therefore, the x-axis of the PIC trajectory represents voltage as well as time, while the y-axis represents current. This is a conventional method to represent PICs in electrophysiological studies. (Dai & Jordan, 2010; Deutsch & Elbasiouny, 2024; Drouillas et al., 2023; Heckman et al., 2008).

**On line 82, the term "trajectory" appears for the first time:** **"If the a-PIC is larger than the d-PIC, the trajectory of the PIC folded from the midline appears counterclockwise; otherwise, a clockwise trajectory of PIC forms." However, this sentence does not clearly define what is meant by "trajectory." It lacks specificity about which trajectory is being referred to and what variables or** **axes are involved.**

**Response:** Thanks for reviewer’s advice. We have rewritten this sentence as “The trajectory of the PIC current shows the evolution of the current vs voltage. This trajectory can be counterclockwise if a-PIC is larger than d-PIC, otherwise a clockwise trajectory of PIC forms.” (Please see lines 81-84)

**In Figure 2, the trajectory is still presented without labeled axes, units, or any clarifying information, while in Figure 3, the y-axis is current and the x-axis is time. Based on my understanding, the term "trajectory" seems to refer to the trajectory of the current in the current-voltage plane, showing how the current evolves. You could phrase it as: "****The trajectory of the PIC current shows the evolution of the current in the current-voltage plane. This trajectory can be counterclockwise if a-PIC is larger than d-PIC..." [continue with the rest of line 83].**

**Response:** Actually, if the reviewer could take a close look at Fig. 2A1, you might find that the calibration bars were clearly marked with units of current (pA), time (s) and voltage (mV). This is a traditional way of representing PICs vs time as well as voltage. The reason is that PICs is a function of both time and membrane potential (voltage), two essential variables to describe the voltage- and time-dependent properties of PICs (Binder et al., 2020; Chen et al., 2022; Cheng et al., 2020; Dai et al., 2009; Dai & Jordan, 2010; Drouillas et al., 2023).

We have added horizontal lines to bi-ramp voltages to show the voltage deference (ΔV) between the onset and offset for clarity in Figure 2A. We also added ramp voltage and ramp current to Fig. 3A and 3B, respectively.

As the reviewer’s suggested, we have changed this sentence to “The trajectory of the PIC current shows the evolution of the current vs voltage. This trajectory can be counterclockwise if a-PIC is larger than d-PIC, otherwise a clockwise trajectory of PIC forms.” (Please see lines 81-84)

**The repeated issue of using the term "trajectory" in the abstract without context, not defining it in the introduction, and leaving figures without proper labeling indicates a lack of clarity that must be addressed before publication. Since "trajectory" is a key concept used throughout the manuscript, it needs to be properly defined and consistently applied to ensure the paper is understandable to a broader audience.**

**Response:** In fact, the “hysteresis” is a professional term used to describe the properties of PICs. However, according to the reviewer’s previous comments on the first version of our manuscript, we replaced it with “trajectory” to simplify the PIC description and to make the PIC property more understandable. In this revised version, we add more definition of 'trajectory' to the Introduction and explain the relationship between the PIC trajectory and neuronal excitability. (Please see lines 81-90)

**II. Quantification of the firing activity: The revised manuscript has significantly improved over the initial version. I appreciated the description of the firing patterns using the parameters Ion, Ioff, Von, and Voff. While these indicate changes in the IV curve, they do not sufficiently explain their connections or their effects on real-world cellular behavior. After this second review, I would like to suggest adding one additional analysis. It seems necessary to quantify the properties of the firing neuron. For instance, I suggest including measurements of the maximum firing frequency under different conditions (gNaP ranging from 1x to 6x, gCaL, and CaL length), the duration of the spiking, and the onset time before spiking begins. Specifically, the maximum frequency could be extracted directly from the peak of the f-I curve, and the duration of the spiking activity could be defined as the** **length of the plateau phase. These parameters would provide a clearer connection to what you introduce regarding** **"various physiological processes in neurons, including the generation and modulation of action potentials, amplification of synaptic current, and regulation of neuronal excitability."**

**Response:** Although we stated in the introduction that "PICs are crucial for various physiological processes in neurons, including the generation and modulation of action potentials, amplification of synaptic current, and regulation of neuronal excitability", this does not mean we should verify these functions of PICs in the present study. Please note we also stated in the introduction: “The purpose of study is to explore the correlation between PIC patterns and discharge types in medullary 5-HT neurons of neonatal mice”. Therefore, the extra new experiments suggested by the reviewer, such as “quantify the properties of the firing neuron”, “measurements of the maximum firing frequency under different conditions (gNaP ranging from 1x to 6x, gCaL, and CaL length) …… might be good for future research but not important to the present study.

**It is striking in Figures 3B, 6B, 7B, and 8B that firing frequency is affected by changes in parameters such as** **gCaL, gNaP, and gCaL distance. For example, the distribution length of gCaL drastically alters the duration of the spiking activity. These parameters likely impact network activity and should be quantified. The fact that Vs can alter spiking duration, frequency, and initiation time could have** **significant effects on network behavior (as shown in Figure 4). Quantifying these aspects would strengthen the connection between the presented data and the physiological relevance of these findings.**

**Response:** Changes in parameters such as gCaL, gNaP, and gCaL distribution could lead to significant alterations in neuronal spiking activity, which could potentially change network activity. However, the present study aims to explore the relationship between PIC patterns and neuronal firing types using a single-cell model which had no neural network involved. The simulation data from a single-cell model are hardly used to interpret the significant effects on network behavior. Therefore, we agree with the reviewer that quantifying relationship between parameters of ion channel and network activity is essential, but this is out of the scope and objectives of the present study. We would like to leave this issue for future study as we did similar work in modeling of spinal networks (Zhang et al., 2022).

**III.Variability in the conductance model: Have you** **considered introducing variability in other conductances, as a real neuron would naturally encounter? While the results are compelling, they may only be accurate for the specific set of parameters chosen. Incorporating variability in additional conductances could provide a more realistic representation of neuronal behavior and strengthen the robustness of your findings. You can replicate your results obtained on this particular set of parameters with a small variability added to your intrinsic parameters (other conductances) and you will compute the mean and the standard deviation of your characteristics (Ion, Ioff, …). It will make the message of Figures 3 to 8 more robust.**

**Response:** First, we would like to thank the reviewer for her meticulous suggestions on our studies. However, we have to frankly say that we did not consider introducing variability in other conductances in the present study, nor did we think this could change the major findings from this study. We believe the core of modeling work is to simplify complex neural system and discover the dominate mechanisms for solving the problems. The model built in this study was a highly simplified 5-HT neuron model, which was good (reliable) for the present study due to the fact that the model had been calibrated with physiological data collected from our experiments (Table 1). However, this model was also limited for exploring the effects of multiple channel modulation (“specific set of parameters”) on the network behavior. Based on our knowledge and experience of more than two decades in locomotor systems, we know that NaT, NaP, Kdr, KCa and CaL are all contributed to regulation of neuronal excitability, but NaP and CaL play a major role in generation of PIC patterns and repetitive firing types in 5-HT neurons (Figure 2). Therefore, the purpose of this study is to explore the how NaP and/or CaL could generate different PIC patterns and how these patterns could produce different firing types in 5-HT neurons. The so-called variability in any other conductance may play a little role in this process.

**Figures in general
- Figure 3: Instead of only showing the trajectory, I recommend replicating Figure 2A and displaying the current trace with the voltage bi-ramp, placing the trajectory in a subpanel to the right of the traces. Similarly, for Figure 3B, I suggest showing the actual firing activity of the neuron model at 100% and 300% of gNaP. I understand your rationale for using percentages to quantify conductance, but indicating 1x and 3x would also be effective. You have added axes everywhere. it is fantastic. But you forgot to add a reference. Add the resting membrane voltage for your voltage traces (example in figure 4C, 1C) and the current same. It is nice to have a scale bar but for example, we also need to see the 0Hz start point on figure 3B.**

**Response:** We appreciate the reviewers' suggestions. We have added the voltage ramp to Figure 3A and 6A as recommended. As the reviewers pointed out, 1X, 3X, and 100%, 300% express the same meaning. However, 1X, 3X … are commonly used to describe the magnification of microscope or multiple thresholds of stimulation current, while the percentage values are commonly used in neurophysiological studies to represent changes in conductance of ionic channels (Dai et al., 2018; Dai et al., 2002; Zhang et al., 2022). For this reason, we prefer to use the percentage format. We have added the initial values of voltage and current for reference to Figures 1-7, the frequency labels to the y-axis in Figures 3B and 6B, and the resting membrane potential of the neurons to Figures 1C, 2B, 4C, 5B, 7C, and 8B.

**- For Figure 8, could you add a plot showing the change in frequency as a function of length, similar to what you did in Figure 7B? Line 536: which f-I curve? example figure 3-B, 6b, you indicate f-seconds. Could you double check all your axes.**

**Response:** In Figure 8B, we aim to elucidate the relationship between changes in dendritic length and the prolongation of neuronal firing. Therefore, the neuronal firing traces represent this relationship much clearer than the instantaneous firing frequency.

The f-I curve mentioned in Line 536 (Line 544 in this revised version) referred to our earlier study, in which we found that increasing the maximum conductance of gCaL altered the f-I relationship of spinal motoneurons in terms of change in slope and intersection of the f-I curve, and an increased excitability of the motoneurons was observed in this case. However, this f-I curve was generated by step currents and quite different to the instantaneous firing frequency generated by bi-ramp currents in the present study.

We have added the stimulus current (ramp current) to Figures 3B and 6B. Thanks to the reviewers for this suggestion.

**Here is the list of typos or small modifications to bring to the text:
* Abstract *
• Line 26: Change "Ionic bases" to "ionic mechanisms."**

**Response:** Thanks for reviewer’s advice. We have changed “ionic bases” to “ionic mechanisms” (Please see lines 26)

**• Line 30-32: Suggested rewording: "PICs in 5-HT neurons were classified into six patterns based on their response to the applied voltage ramp. Three firing activities were defined according to their response to the current ramp injection."**

**Response:** Thanks for reviewer’s advice. We have revised the sentence to: “PICs in 5-HT neurons were classified into six patterns based on their current trajectory induced by bi-ramp voltage and activation voltage, while repetitive firings were categorized into three types according to their response to bi-ramp current injection.” (Please see lines 30-33)

**• Line 41: For clarity, consider: "This study provides insights into the ionic mechanisms underlying the generation of multiple PIC patterns..."**

**Response:** Thanks for reviewer’s advice. We have revised this sentence. (Please see lines 41-43)

*** Introduction *
Consider emphasizing this result in the abstract, as it is quite significant. If it has already been included, it may benefit from clearer phrasing to ensure the importance of the finding is effectively conveyed**

**Response:** Thanks for reviewer’s advice. We have revised the conclusion in the Abstract. (Please see lines 41-43)

*** Results *
• Line 136: In Table 1, subscript 'm' in Eₘ and in Rₗₙ.**

**Response:** Thanks for reviewer’s advice. We have made the revisions. (Please see lines 144)

**• Line 176: Add axis labels for the trajectory—current on the y-axis (I) and voltage on the x-axis (V). This is still unclear.**

**Response:** We have rewritten the sentence as “The PICs had only a-PIC with counterclockwise trajectory and ΔV > 0, current trajectory recorded by bi-ramp voltage from -70 mV to 50 mV (bottom).” (Please see lines 183-185)

**• Line 183: I like the horizontal dotted lines for Ion and Ioff. Add them to Panel A for Von and Voff as well.**

**Response:** In response to the reviewers' suggestion, we have added a horizontal dotted lines for V_onset_ and V_offset_ to Panel A. (Please see Figure 2A)

• **Line 232: The x-axis in the trajectory plot is incorrect. The trajectory is the line on the I-V plane.**

**Response:** For clarity, we have added the voltage ramps that induced the PICs to Figure 3A. (Please see Figure 3A)

**• Line 234: In Figures 3A-B, add the dot and triangle from Figure 3C to directly show Von, Voff, Ion, and Ioff. Line 234: Could you add on Fig3A-B the dot and the triangle associated with Fig3C. We can directly see the Von, Voff, Ion and Ioff.**

**Response:** We have used closed circle and triangles in Figures A and B to represent the V_onset_, V_offset_, I_onset_, and I_offset_. (Please see Figure 3A and B)

**• Line 234: In Figures 3A-B, add the dot and triangle from Figure 3C to directly show Von, Voff, Ion, and Ioff.**

**Response:** Please refer to the response to the above question.

**• Line 240: In Figure 3F, you describe a slight change with a slope of 0.00002, but in Figure 4G, you say no change with a slope of 0.007. These descriptions are inconsistent.**

**Response:** Thanks for the reviewers' comments. We have rewritten these two sentences as “However, the ratio of amplitude (d-PIC/a-PIC) did not change (Fig. 3F).” and “These results indicated that increasing the V_S_ of NaP had little effect on the counterclockwise trajectory (Fig. 4F and G) of the PICs and the ΔV (Fig. 4D), nor did it change the firing type of the neuron (Fig. 4E).” (Please see line 234 and 265)

**• Line 264 (Figure 4): Follow a consistent convention for the y-axis labels (m_{in,NaP} ) in the equation and throughout the text. In Panel A, there are two different conventions. Add "( m_inf,NaP)" in the legend.**

**Response:** We have named the state variables of the ion channels in both Figures 4&7 and throughout the manuscript.

**• Line 266: Use "bi-ramp" or "ramp" consistently throughout the paper.**

**Response:** We have replaced "ramp" with "bi-ramp" throughout the manuscript.

**• Line 270: Color-code the triangle and circle in red and orange to match Panel A.**

**Response:** Figure 4D shows the trend of V_onset_ and V_offset_ as a function of V_S_, without emphasizing specific numerical values. Therefore, we have used uniform black markers to improve readability.

*** Discussion *
• Line 596: The term "hysteresis" is still present.**

**Response:** We have replaced "hysteresis" by "trajectory". (Please see lines 84)

**• Line 603: Consider moving this section to the introduction. If this is not your result, it would help readers understand why you chose "trajectory" as a key factor for analysis throughout the paper.**

**Response:** We have revised this section and added it to the Introduction. (Please see lines 84-90)

**• Line 609, 611, 618: Avoid repeating "in this present study" within such close proximity.**

**Response:** We have revised the sentences in line 629 and 631. Thanks.

**• Line 613: Include the frequency, duration, and spiking initiation time in the discussion (refer to general comment II).**

**Response:** We have added discussion about the relationships between PICs and neuronal firing frequency, duration of neuronal firing, spiking initiation time to the Discussion section. (Please see lines 609-611)

*** Methods*
• Line 787: Consider adding the** **full name of the channel each time. For example, in line 786 you use "m," and again in line 802. It’s standard in computational modeling papers to follow the convention of using activation variables followed by the ion channel notation. The same applies m_inf,NaP … etc. This was mentioned in the previous review as well: "The same notation 'm' is used as in line 681. I suggest adding subscripts to differentiate the currents described, such m_Na, h_Na, and m_NaP**

**Response:** Thanks for reviewer’s advice. We have added the full name of the activation variables to the formula.

**• I would like to emphasize that the codes and the models are available in the github.**

**Response:** We have confirmed that both the codes and the models are available in the github.

**Reference:**

Binder, M. D., Powers, R. K., & Heckman, C. J. (2020). Nonlinear Input-Output Functions of Motoneurons. *Physiology (Bethesda)*, *35*(1), 31-39. <https://doi.org/10.1152/physiol.00026.2019>

Chen, K., Ge, X., & Dai, Y. (2022). Cholinergic modulation of persistent inward currents is mediated by activating muscarinic receptors of serotonergic neurons in the brainstem of ePet-EYFP mice. *Experimental Brain Research*, *240*(4), 1177-1189. <https://doi.org/10.1007/s00221-022-06322-w>

Cheng, Y., Zhang, Q., & Dai, Y. (2020). Sequential activation of multiple persistent inward currents induces staircase currents in serotonergic neurons of medulla in ePet-EYFP mice. *J Neurophysiol*, *123*(1), 277-288. <https://doi.org/10.1152/jn.00623.2019>

Dai, Y., Carlin, K. P., Li, Z., McMahon, D. G., Brownstone, R. M., & Jordan, L. M. (2009). Electrophysiological and pharmacological properties of locomotor activity-related neurons in cfos-EGFP mice. *J Neurophysiol*, *102*(6), 3365-3383. <https://doi.org/10.1152/jn.00265.2009>

Dai, Y., Cheng, Y., Fedirchuk, B., Jordan, L. M., & Chu, J. (2018). Motoneuron output regulated by ionic channels: a modeling study of motoneuron frequency-current relationships during fictive locomotion. *J Neurophysiol*, *120*(4), 1840-1858. <https://doi.org/10.1152/jn.00068.2018>

Dai, Y., Jones, K. E., Fedirchuk, B., McCrea, D. A., & Jordan, L. M. (2002). A modelling study of locomotion-induced hyperpolarization of voltage threshold in cat lumbar motoneurones. *J Physiol*, *544*(2), 521-536. <https://doi.org/10.1113/jphysiol.2002.026005>

Dai, Y., & Jordan, L. M. (2010). Multiple patterns and components of persistent inward current with serotonergic modulation in locomotor activity-related neurons in Cfos-EGFP mice. *J Neurophysiol*, *103*(4), 1712-1727. <https://doi.org/10.1152/jn.01111.2009>

Deutsch, A. J., & Elbasiouny, S. M. (2024). Dysregulation of persistent inward and outward currents in spinal motoneurons of symptomatic SOD1-G93A mice. *J Physiol*, *602*(15), 3715-3736. <https://doi.org/10.1113/jp286032>

Drouillas, B., Brocard, C., Zanella, S., Bos, R., & Brocard, F. (2023). Persistent Nav1.1 and Nav1.6 currents drive spinal locomotor functions through nonlinear dynamics. *Cell Rep*, *42*(9), 113085. <https://doi.org/10.1016/j.celrep.2023.113085>

Heckman, C. J., Johnson, M., Mottram, C., & Schuster, J. (2008). Persistent inward currents in spinal motoneurons and their influence on human motoneuron firing patterns. *Neuroscientist*, *14*(3), 264-275. <https://doi.org/10.1177/1073858408314986>

Zhang, Q., Cheng, Y., Zhou, M., & Dai, Y. (2022). Locomotor Pattern and Force Generation Modulated by Ionic Channels: A Computational Study of Spinal Networks Underlying Locomotion. *Front Comput Neurosci*, *16*, 809599. <https://doi.org/10.3389/fncom.2022.809599>
